# Seizure control by adding on other anti-seizure medication on seizure during levetiracetam administration in patients with glioma-related epilepsy

**DOI:** 10.1186/s12885-023-11273-8

**Published:** 2023-09-11

**Authors:** Etsuko Yamamoto Hattori, Yoshiki Arakawa, Yohei Mineharu, Keiko Furukawa, Yukinori Terada, Yukihiro Yamao, Masahiro Tanji, Takayuki Kikuchi, Susumu Miyamoto

**Affiliations:** 1 Department of Neurosurgery, Kyoto University Graduate School of Medicine, 54 Kawaharacho, Shogoin, Sakyo-ku, Kyoto City, Kyoto 606-8507 Japan; 2 Cancer Center, Kyoto University Hospital, Kyoto, Japan

**Keywords:** Antiseizure medication, Levetiracetam, Glioma-related epilepsy, Seizure occurrence, Increasing dose

## Abstract

**Background:**

Epilepsy is a major symptom in patients with glioma. Levetiracetam (LEV) is recognized as a first-line treatment for glioma-related epilepsy. Increasing the LEV dose is allowed into patients with seizure occurrence against its initial dose. However, the therapeutic efficacy of increasing the LEV dose in response to seizure occurrence remains unclear.

**Methods:**

We retrospectively analyzed 236 glioma patients who were treated with antiseizure medications (ASMs) internally at our institute between September 2010 and December 2017. Of these, the analysis focused on 156 patients treated with LEV who had a clear history of administration.

**Results:**

Seizure occurrences were observed in 21 of 75 patients (26.7%) who received LEV as first-line therapy and in 33 of 81 patients (40.7%) who received LEV as non-first-line treatment. The seizure control rate for seizure occurrence with LEV as first-line treatment was significantly higher in patients treated with addition of other ASMs (72.7%) than in those treated with increasing dose of LEV (20.0%) (*p* = 0.016). The seizure control rate for seizure occurrence with LEV as non-first-line treatment did not differ significantly between patients with addition of other ASMs (58.3%) and those treated with increasing dose of LEV (47.6%) (*p* = 0.554).

**Conclusions:**

Adding other ASMs was more effective than increasing the LEV dose for seizure control in patients treated with LEV as first-line treatment, but they demonstrated comparable efficacy in patients treated with LEV as non-first-line treatment.

**Supplementary Information:**

The online version contains supplementary material available at 10.1186/s12885-023-11273-8.

## Background

Around 15–50% of patients with glioma reportedly experience epilepsy as the first clinical symptom [1], and 60–85% of patients with low-grade glioma experience epilepsy during their lifespan [2–4]. Surgical removal of the tumor is effective to control tumor-associated epilepsy [5]. However, not all lesions are resectable, and antiseizure medications (ASMs) play an important role in this setting for seizure management.

In addition to conventional ASMs, second-generation ASMs have become widely available, including levetiracetam (LEV), lamotrigine (LTG), topiramate (TPM) and gabapentin (GBP). Third-generation drugs such as lacosamide (LCM) and perampanel (PER) have also recently become available. Newly developed ASMs involve different mechanisms of action, and have fewer adverse events and drug-drug interactions [6, 7]. Although seizure control rates in tumor-related epilepsy have improved with the advent of these new ASMs [7], many patients still cannot achieve seizure control with one drug alone and need treatment with multiple drugs or increased doses of ASMs above the regular dosage. Although many reports have examined the outcomes of glioma-related epilepsy with new-generation ASMs [7–13], evidence for the management of recurrent seizures, including details of seizure control rates and adverse events, remains lacking.

LEV is the most widely used ASM for patients with brain tumor-related epilepsy [14, 15]. LEV can be started at the maintenance dosage of 500 mg b.i.d., and the dose can be increased to 1500 mg b.i.d. according to the clinical condition [16]. However, the therapeutic efficacy of increasing the dose of LEV is unclear in patients with glioma-related epilepsy. This study therefore investigated the seizure rate between patients under LEV medication who underwent dose increase compared to those who added other ASMs. We also analyzed adverse events associated with LEV and other ASMs to clarify the optimal management of glioma-related epilepsy.

## Methods

### Study design and data collection

This study was approved by the Ethics Committee, Kyoto University Graduate School and Faculty of Medicine (approval number: R1461) and in accordance with the guidelines of the Declaration of Helsinki. We retrospectively analyzed the medical records of glioma patients who started treatment with ASMs at Kyoto University Hospital between September 2010 and December 2017. LEV began to be covered by insurance in Japan in September 2010. Demographic characteristics, pathological diagnosis, location of glioma, outcome of glioma, diagnosis of epilepsy, types and dosages of ASMs, seizure-free period, adverse events, and changes in the types and dosages of ASMs were recorded. The primary outcome was the seizure control rate, which was defined as seizure-free condition from the start of follow-up or change of ASM until the end of follow-up. The determination that a seizure had occurred was based on adjustment of ASM. The analysis included patients who received ASMs prophylactically. ASM focused on oral administration. When oral administration of LEV became difficult, especially in the perioperative period, the patient was switched to intravenous infusion. Generally, it is reported that the switch to intravenous infusion takes about 2 days [17], and our hospital followed this policy while prioritizing the patient’s condition. With regard to LEV, the difference in perioperative administration route was considered acceptable because the maximum plasma concentration (Cmax) and area under the concentration-time curve (AUC) were equivalent between intravenous and oral administration and are considered to be bioequivalent [18, 19].

### Statistical analysis

Statistical analysis was performed using GraphPad Prism software (version 9.1.0) or JMP Pro statistical software (version 14.0.0; SAS Institute Inc., Cary, NC). Seizure control rate was compared using the chi-square test, Fisher’s exact test and Kaplan-Meier method. Factors associated with the seizure control rate of ASMs (e.g., age, sex, tumor location, treatment methods and pathological diagnosis (WHO grade)) were analyzed by Student’s t-test or the chi-square test or Cox proportional hazards model as appropriate.

## Results

### Characteristics and ASM usage in patients with glioma

A total of 278 patients were diagnosed with intracranial glioma in our hospital between 2010 and 2017. Characteristics of the study population are shown in Table 1. Mean age at diagnosis was 49.9 years (range, 6–90 years). Median duration of follow-up was 20 months (range, 1–135 months). The most common tumor location was the frontal lobe, followed by the temporal lobe. Some patients showed multiple lesions. Glioblastoma was the most common pathological diagnosis.

**Table 1:** Demographic and pathological characteristics of the study population

	Total	With ASM	Without ASM
Number of patients	278	236	42
Female; n (%)	112 (40.0)	94 (39.8)	18 (42.9)
Age, years; mean (SD)	49.9 (19.3)	49.9 (18.3)	49.9 (24.1)
Location			
Frontal lobe	103	96	7
Temporal lobe	74	68	6
Parietal lobe	32	29	3
Other locations	50	27	23
Multiple lesions	19	16	3
Pathological diagnosis			
Glioblastoma	120	107	13
Anaplastic astrocytoma	49	41	8
Anaplastic oligodendroglioma	11	10	1
Diffuse astrocytoma	43	38	5
Oligodendroglioma	18	17	1
Oligoastrocytoma / anaplastic oligoastrocytoma (~ 2015)	13	12	1
Glioma of undetermined pathology	24	11	13

ASM, antiseizure medication; SD, standard deviation

Of the initial 278 patients, 236 patients had been prescribed ASMs. ASMs were administered prophylactically in 101 patients, started after the seizure in 124 patients, and the timing of administration was unknown in 11 patients. LEV was the most common ASM (n = 75, 31.8%) used as first-line treatment, followed by zonisamide (ZNS) (n = 64, 27.1%) and valproic acid (VPA) (n = 37, 15.7%) (Table 2). Patients who had been administered two ASMs by a previous hospital were counted for each ASM, so the total number of patients administered each ASM exceeded the number of patients.

**Table 2:** Initial ASM administered in 236 patients with glioma

	Patient number (percentage of total) *
LEV	75 (31.8)
ZNS	64 (27.1)
VPA	37 (15.7)
CBZ	17 (7.2)
PHT	17 (7.2)
CLB	4 (1.7)
LTG	4 (1.7)
GBP	4 (1.7)
PB	3 (1.3)
Unknown**	18 (7.6)

* Patients who had been administered multiple ASMs at the previous hospital were counted under each ASM (VPA and ZNS, 2 patients; LEV and GBP, 2; LEV and VPA, 2; PB and PHT,1; LTG and ZNS, 1; PHT and ZNS, 1)

** Patients classed as “unknown” were those who had been treated at other hospitals for a long time and had an uncertain medication history

ASM, antiseizure medication; LEV, levetiracetam; ZNS, zonisamide; VPA, valproic acid; CBZ, carbamazepine; PHT, phenytoin; CLB, clobazam; PER, perampanel; LTG, lamotrigine; GBP, gabapentin; PB, phenobarbital.

**Fig. 1:**
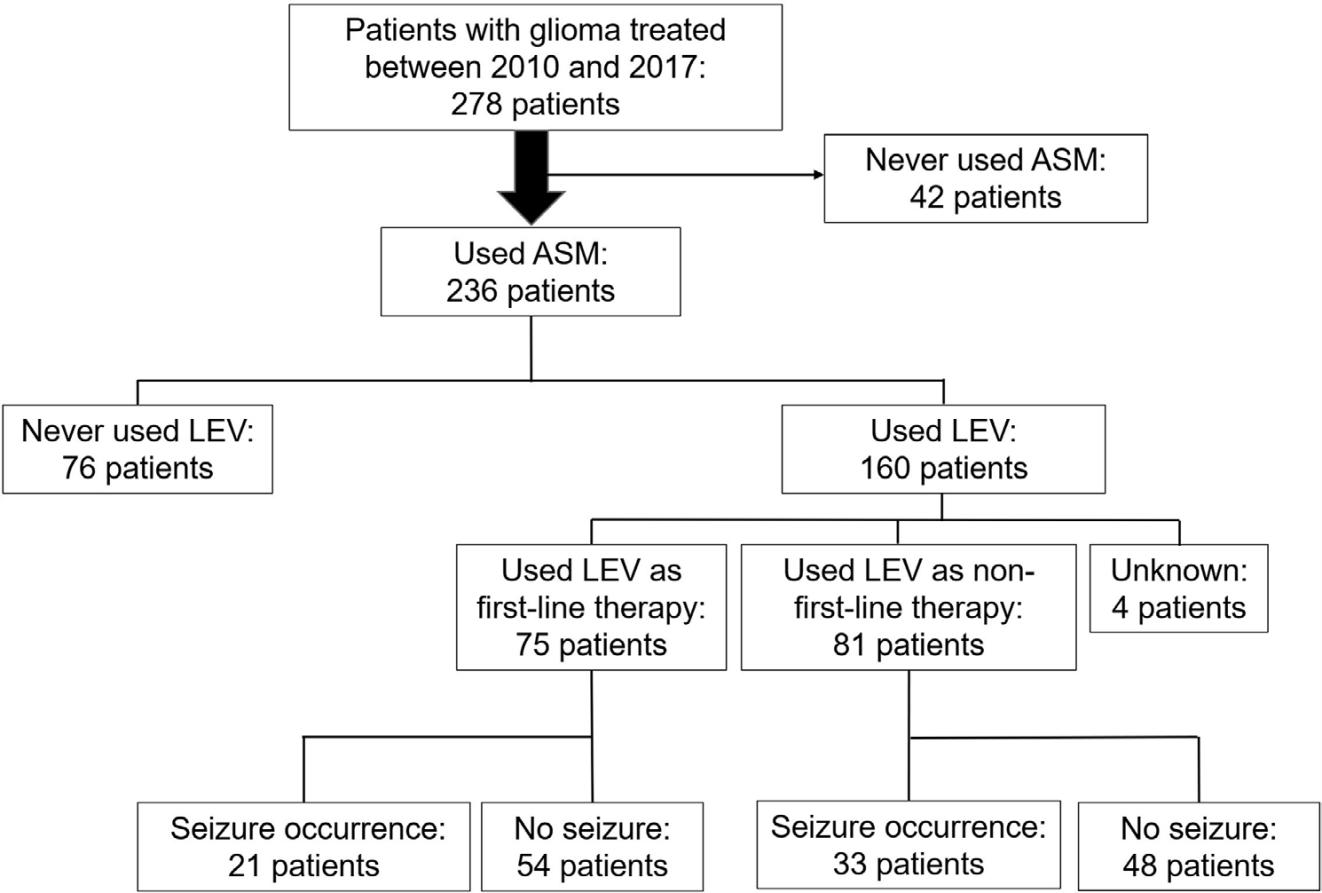
Flow chart for participation in the study. Among 278 patients with glioma who were treated at our hospital between 2010 and 2017, we excluded 42 patients who had never used ASMs. Of the remaining 236 patients, 160 patients had used LEV and were analyzed in this study, except for 4 patients from whom a detailed history of medication could not be traced in the medical records. We divided these 156 patients into two groups: one group of 75 patients who had received LEV as first-line treatment; and the other group of 81 patients who had received LEV as non-first-line treatment

Next, we divided the study population according to ASM usage (Fig. 1). As LEV was the most frequently used ASM, we stratified patients according to the usage of LEV. Seventy-five patients had received LEV as the first-line treatment, and 81 patients had received LEV as the non-first-line treatment. The median initial dose of LEV in both groups was 1000 mg/d. Four patients had already received LEV in the previous hospital and their medical records were unclear regarding whether LEV was prescribed as the first-line or non-first-line treatment. Among the 156 patients excluding these four, seizure occurrence, including seizure or focal awareness seizure (FAS), was seen after LEV treatment in 55 patients (35.5%). Of these patients, 14 (25.5%) achieved gross total resection of their gliomas. Of the 101 patients who did not have a seizure relapse, 24 (23.8%) achieved gross total resection of their gliomas, and there appeared to be no association between glioma resection status and seizure relapse (*p* = 0.814).

The 55 patients who had seizures after receiving LEV included 21 patients (26.7%) who received LEV as first-line treatment and 33 patients (40.7%) who received LEV as non-first-line treatment (Fig. 1). As the seizure occurrence rate was slightly, but not significantly, higher in patients with LEV as non-first-line treatment than in those with LEV as first-line treatment, we analyzed these two patient groups separately.

### Seizure control by increasing dose of LEV in patients who received LEV as first-line treatment

**Fig. 2:**
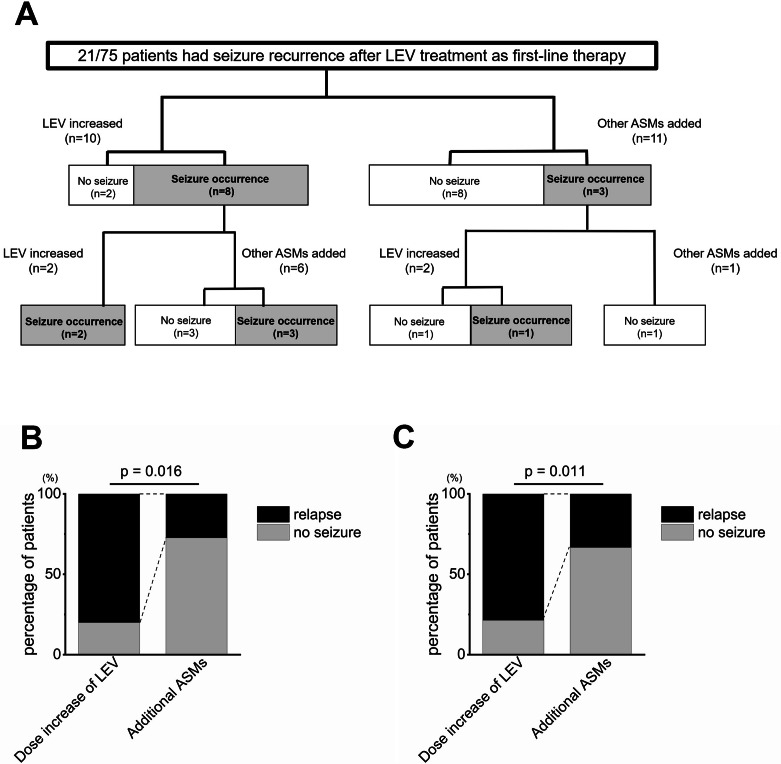
Significantly better seizure control by adding other ASMs rather increasing the LEV dose in patients who received LEV as first-line treatment. **A**: Among the 75 patients who had received LEV as the first-line ASM, 21 experienced seizure occurrences. Ten of these were treated with an increased dose of LEV and 11 were treated with addition of other ASMs. **B**: Seizure control rate after the first ASM addition was significantly higher in patients treated by adding other ASMs (8/11, 72.7%) than in those treated by increasing dose of LEV (2/10, 20.0%; *p* = 0.016). **C**: When all events up to seizure occurrences after the second ASM addition were included, the seizure control rate was significantly higher with the addition of other ASMs (12/18, 66.7%) than with an increased dose of LEV (3/14, 21.4%; *p* = 0.011)

Among the 75 patients who received LEV as first-line treatment, 21 patients experienced seizure. Tumor location and WHO grade had no effect on seizure incidence (Cox proportional hazards model, Wald test; p = 0.894, 0.161). Of these, 10 patients were treated by increasing the dose of LEV, while the remaining 11 patients were treated by adding other ASMs (Fig. 2A). The median LEV increase dose was 1000 (500–1000) mg. ASMs added to LEV was mostly PER, clobazam (CLB) and VPA (Supplemental Table 1). No significant difference in clinical background was seen between groups with respect to sex, age, tumor location, treatment methods or pathological diagnosis (Table 3).

**Table 3:** Comparison between increased LEV dose and addition of other ASMs to prevent seizure recurrence in patients who received LEV as first-line treatment

	LEV dose increased	Other ASM added	*p* value
Number	10	11	
Female, n (%)	2 (20.0)	6 (54.5)	0.104
Age, mean (SD)	45.0 (18.4)	46.2 (20.4)	0.892
Location, n			
Frontal lobe	3	6	0.231
Temporal lobe	6	2	
Parietal lobe	0	1	
Other locations	1	2	
Pathological diagnosis, n			
Glioblastoma	3	5	0.761
Anaplastic astrocytoma	4	3	
Anaplastic oligodendroglioma	0	1	
Diffuse astrocytoma	1	1	
Oligodendroglioma	1	0	
Oligoastrocytoma / anaplastic oligoastrocytoma (~ 2015)	1	1	
Surgery, n			0.466
Gross total resection	3	5	
Others with resection	7	6	
Radiotherapy, n	9	10	0.943
Chemotherapy, n	9	9	0.593
Primary seizure relapse, n (%)	8 (80.0)	3 (27.3)	0.016
Secondary seizure relapse, n (%)	3/4 (75.0)	3/7 (42.9)	0.303
Primary + Secondary, n (%)	11/14 (78.6)	6/18 (33.3)	0.011

ASM, antiseizure medication; LEV, levetiracetam; SD, standard deviation.

Seizure control rate after the first ASM addition was significantly higher in patients treated by adding other ASMs (8/11, 72.7%) than in those treated by increasing the dose of LEV (2/10, 20.0%; chi-square test *p* = 0.016, Fisher’s exact test *p* = 0.030 ) (Fig. 2B). When all events up after the second ASM addition were included, the seizure control rate was significantly higher with the addition of other ASMs (12/18, 66.7%) than with increasing dose of LEV (3/14, 21.4%; chi-square test *p* = 0.011, Fisher’s exact test *p* = 0.016) (Fig. 2C). However, when analyzed using the Kaplan-Meier method, including the follow-up period, the addition of other ASMs tended to suppress seizures better than increasing the dose of LEV, but the difference was not statistically significant (*p* = 0.163) (Supplementary Fig. 1). For a total of 18 cases who added other ASM in the first and second ASM changes, the LEV dose was unchanged in 15 cases, reduced in 1 case, and discontinued in 2 cases, with the majority of patients not adjusting their LEV dose.

### Seizure control by increasing dose of LEV in patients who received LEV as non-first-line treatment

**Fig. 3:**
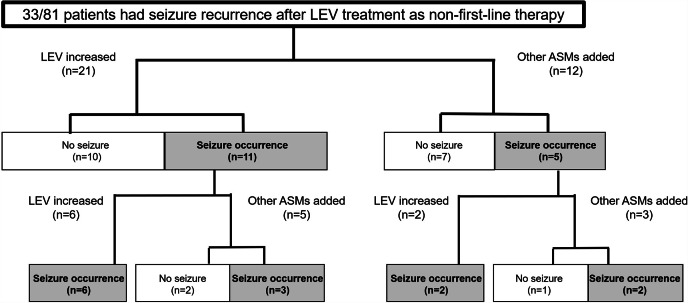
Difference in seizure control between adding other ASMs and increasing the LEV dose in patients received LEV as non-first-line treatment. Among the 81 patients who received LEV as a non-first-line ASM (and thus had received at least two ASMs, including LEV), 33 experienced seizure occurrences. Twenty-one patients were treated with an increased dose of LEV and the remaining 12 patients were treated with addition of other ASMs. Seizure control rate after the first ASM addition tended to be higher in patients with addition of other ASMs (7/12, 58.3%) than in those treated with an increased dose of LEV (10/21, 47.6%; *p* = 0.554). When all events after the second ASM addition were included, seizure control rates did not differ significantly between addition of other ASMs (10/20, 50.0%) and increasing dose of LEV (10/29, 34.5%; *p* = 0.277)

Among the 81 patients who received LEV as non-first-line treatment, 33 patients experienced seizure occurrence. Tumor location and WHO grade had no effect on seizure incidence (Cox proportional hazards model, Wald test; p = 0.789, 0.871). Of the 33 patients, 21 patients were treated with an increased dose of LEV, and the remaining 12 patients had received addition of other ASMs (Fig. 3). The median LEV increase dose was 500 (250–1250) mg. The ASMs added to LEV were CLB, PER, and ZNS (Supplemental Table 1). No significant differences in background or pathological characteristics including age, sex, tumor location, treatment methods or pathological diagnosis were seen between the two groups (Table 4).

**Table 4:** Comparison between increased LEV dose and addition of other ASMs to prevent seizure recurrence in patients who received LEV as non-first-line treatment for glioma-related epilepsy

	LEV dose increased	Other ASMs added	*p* value
Number	21	12	
Female, n (%)	7 (33.3)	4 (33.3)	1
Age, mean (SD)	41.6 (16.4)	47.8 (15.9)	0.301
Location, n			0.338
Frontal lobe	9	6	
Temporal lobe	7	1	
Parietal lobe	3	4	
Other locations	2	1	
Pathological diagnosis, n			0.719
Glioblastoma	8	6	
Anaplastic astrocytoma	3	1	
Anaplastic oligodendroglioma	0	0	
Diffuse astrocytoma	6	3	
Oligodendroglioma	2	0	
Oligoastrocytoma / anaplastic oligoastrocytoma (~ 2015)	2	1	
Others	0	1	
Surgery, n			0.865
Gross total resection	4	2	
Others with resection	17	10	
Radiotherapy, n	19	11	0.909
Chemotherapy, n	18	9	0.443
Primary seizure occurrence, n (%)	11 (52.4)	5 (41.7)	0.554
Secondary seizure occurrence, n (%)	8/8 (100)	5/8 (62.5)	0.055
Primary + Secondary, n (%)	19/29 (65.5)	10/20 (50.0)	0.277

ASM, antiseizure medication; LEV, levetiracetam; SD, standard deviation.

Seizure control rate after the first ASM addition was not statistically significantly different between patients with addition of other ASMs (7/12, 58.3%) and those who treated with an increased dose of LEV (10/21, 47.6%; chi-square test *p* = 0.554, Fisher’s exact test *p* = 0.721). Similarly, when all events after the second ASM addition were included, seizure control rates did not differ significantly between addition of other ASMs (10/20, 50.0%) and increasing dose of LEV (10/29, 34.5%; chi-square test *p* = 0.277, Fisher’s exact test *p* = 0.377) (Fig. 3). When the Kaplan-Meier method was used, there was no statistically significant difference in seizure control rates between the other ASM addition group and the LEV dose increase group (*p* = 0.602) (Supplementary Fig. 2). For a total of 20 patients who added other ASM in the first and second ASM changes, the LEV dose was unchanged in 12 cases, reduced in 1 case, discontinued in 1 case, and increased in 6 cases. Although most of the cases did not adjust the LEV dose, it was characteristic that some cases increased the LEV dose at the same time as the addition of other ASM, compared to the patients who received LEV as first-line treatment.

### Adverse events of ASM treatment

The incidence of adverse events was examined in 236 patients who had previously received ASM, counting those with adverse events noted in the medical record, regardless of the time between the start of ASM and the occurrence of the adverse event. 85 patients (36.0%) had adverse events (87 cases; 2 patients had adverse events to multiple drugs). In response to adverse events, 34 cases (39.1%) discontinued ASM, 33 cases (37.9%) changed the ASMs, 11 cases (12.6%) reduced the dosage, and 9 cases (10.3%) did not change anything. This included patients with poor compliance. Six patients (2.5%) were determined to be poor ASM compliant based on medical records, one patient had no change in ASM, and five patients discontinued or reduced ASM at their own decision. Among the patients who discontinued or reduced ASM, 2 patients had seizures and were switched to other ASM.

Next, adverse events associated with each ASM were reviewed (Supplemental Table 2). For some patients receiving multiple medications, it was difficult to determine which ASM was responsible for the adverse event. In such patients, adverse events were counted for all prescribed ASMs. The number of adverse events for LEV was 34 (21.3%) out of 160 patients. Other ASMs also generally had an incidence rate of 10–30%, and there was no significant difference in the incidence of adverse events by ASM (*p* = 0.279).

We then analyzed adverse events associated with increasing dose of LEV or adding other ASMs. In patients with an ASM reduced in dose or withdrawn due to an adverse event within 1 month after prescription, the ASM was defined as the causative drug. Of the 43 patients with an increase in the dose of LEV, 1 patient (2.3%) experienced an adverse event after the dose increase. Of the 50 patients with addition of other ASMs, 2 patients (4.0%) experienced adverse events after the dose increase (Table 5). There was no statistically significant difference between the two groups (*p* = 0.649). Compared to other ASMs, the incidence of LEV adverse events by dose increasing or addition of other ASMs was not statistically higher (other ASM added: *p* = 0.927, dose increased: *p* = 0.974).

**Table 5:** Adverse events associated with increasing dose of LEV dose or addition of other ASMs

Number of patients with adverse events / Total number of each ASM added or increased patients	LEV	ZNS	VPA	CBZ	PHT	CLB	PER	LTG	*p* value
Other ASM added	2 / 50 (4.0)	5 / 71 (7.0)	2 / 45 (4.4)	0 / 24 (0)	1 / 24 (4.2)	0 / 18 (0)	0 / 0 (0)	0 / 7 (0)	0.927
Dose increased	1 / 43 (2.3)	1 / 13 (7.7)	1 /12 (8.3)	0 / 8 (0)	0 / 4 (0)	1 / 10 (10.0)	1 / 5 (20.0)	1 / 6 (16.7)	0.974

ASM, antiseizure medication; LEV, levetiracetam; ZNS, zonisamide; VPA, valproic acid; CBZ, carbamazepine; PHT, phenytoin; CLB, clobazam; PER, perampanel; LTG, lamotrigine

*Numbers in parentheses represent the percentage of adverse events that occurred relative to the number of patients added or dose increased to each ASM.

## Discussion

LEV is reportedly highly effective and well-tolerated for tumor-related epilepsy [14, 20–22] LEV was the most common ASM used for patients with glioma in our hospital, and was chosen for first-line treatment. This study was a retrospective study, and ASM was selected subjectively by the physician in charge. LEV has the pharmacological characteristics of being eliminated unchanged by the kidney, having no drug-drug interactions, and requiring no treatment titrated up from the initial dose [23]. Since glioma patients are more likely to be taking anticancer drugs and multiple medications characteristic of the elderly, we assume that LEV, which is easy to use, was often chosen as the first-line ASM in our hospital. A simple comparison of seizure control rates by first-line drugs showed that LEV performed well, but no significant difference was evident between ASMs. With LEV, 1- and 2-year seizure-free survival rates were 60.0% and 48.3%, showing that seizure relapse tends to occur within 1 year. Seizures can still recur after 1 year, but the risk becomes much lower. Seizure control during the early phase seems important, and while some reports have shown that administration of an increased dose of LEV is effective and tolerable to prevent seizure occurrence [24, 25], other reports have failed to show significant differences in seizure control rate of LEV between normal and higher-dose ranges [26, 27].

Recently, the development of new-generation anticonvulsants has enabled us to add ASMs because of the high tolerability with negligible drug-drug interactions [28]. For example, PER and LCM are recognized as having little influence on or from other drugs, including previously administered ASMs [29, 30]. In this study, PER was most frequently used as an additional ASM, although LCM was not listed because it only became available in Japan in 2010. We thus aimed to compare the effectiveness of different strategies for seizure control, namely increasing the dose of LEV or adding other ASMs. The addition of other ASMs was mostly in the form of add-on therapy, although some patients were changed to other ASMs by tapering off LEV. Our data showed that sufficient seizure control was not achieved by increasing the dose of LEV and that add-on therapy provided better seizure control in patients with LEV as first-line treatment. Further, add-on therapy was well tolerated with no significant increase in adverse events as compared to increasing the dose of LEV. Lee et al. also compared the effectiveness of increasing the LEV dose and add-on therapy with LCM for patients with epilepsy, showing that add-on LCM was no less effective than increasing the LEV dose, while resulting in significantly fewer adverse events [31]. On the other hand, Brodie et al. compared the effectiveness of add-on therapy according to ASMs and showed that all types of ASMs had comparable effects [32]. Although the types of ASMs added to LEV varied in our cohort, the results suggest that add-on therapy is a reasonable option for recurrent seizures regardless of the type of ASMs. Rather than adjusting with a single LEV agent, it was preferable to administer multiple agents with different mechanisms of action.

In our cohort, better seizure control from addition of other ASMs was identified in patients treated with LEV as first-line treatment, but the effect was reduced in patients treated with LEV as non-first-line treatment. The former patients were mainly treated with LEV monotherapy and their seizures were well controlled, whereas the latter patients were treated with dual or triple ASMs because of refractory seizures. Still, addition of other ASMs was not inferior to increasing dose of LEV and adverse events were also tolerable. As for increasing the LEV dose, high blood levels of LEV might increase the incidence of adverse events [27, 33]. However, this study showed that adverse event rates in patients receiving increasing dose of LEV were low (1/43, 2.3%), suggesting that increasing dose of LEV remains an important option for the treatment of refractory epilepsy. The treatment strategy should be selected depending on the clinical condition of patients.

Several limitations must be acknowledged in this study. First, this was a retrospective study at a single institute and the number of eligible patients was small. The statistical power may have been insufficient, and the clinical background may not have been well-balanced between treatment groups. Second, adverse events were collected from medical records, and this method is susceptible to recall bias and reporting bias. In fact, LEV is reportedly associated with psychiatric symptoms such as aggression or anxiety [34], but this event was rarely seen in our cohort. Further study is therefore needed to validate our findings. Third, some patients in this study underwent chemoradiotherapy and adverse events may thus have been affected by the radiation dose or chemotherapy regimen, especially for cases involving cytopenia, anorexia, and hepatic dysfunction. Fourth, not all of the patients in this study had an electroencephalogram, and they were only considered to have had seizures if there was a clear description of a seizure in the medical record and the ASMs were changed. It is possible that the nonconvulsive status epilepticus was overlooked. Furthermore, the seizure types of the target patients were various, and the differences in the effects of ASMs according to the seizure type was not considered.

## Conclusions

This study indicated that both increasing the dose of LEV and adding other ASMs are effective and tolerable strategies for addressing seizure occurrence in patients with glioma-related epilepsy. Adding other ASMs was more effective than increasing the LEV dose for seizure control in patients treated with LEV as first-line treatment. In patients treated with LEV as non-first-line treatment, the efficacies of adding other ASMs and increasing the LEV were not different for seizure control. Taken together, addition of other ASMs may represent an effective treatment option to prevent further seizures among patients with glioma-related epilepsy receiving LEV.

### Electronic supplementary material

Below is the link to the electronic supplementary material.

Supplementary Material 1

Supplementary Material 2

Supplementary Material 3

Supplementary Material 4

## Data Availability

The datasets used and analysed during the current study are available from the corresponding author on reasonable request.

## List of abbreviations

ASM: Antiseizure medication
CLB: Clobazam
FAS: Focal awareness seizure
GBP: Gabapentin
LCM: Lacosamide
LEV: Levetiracetam
LTG: Lamotrigine
PB: Phenobarbital
PER: Perampanel
SD: Standard deviation
TPM: Topiramate
VPA: Valproic acid
ZNS: Zonisamide

## Declarations

### Previous presentations

No portion of this paper has been presented previously.
