# Supplementary figures and images for: Seizure control by adding on other anti-seizure medication on seizure during levetiracetam administration in patients with glioma-related epilepsy
Source: BMC Cancer. 2023 Sep 11;23:849. doi: 10.1186/s12885-023-11273-8 (PMC10496310; doi:10.1186/s12885-023-11273-8)

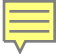

Supplementary Figure 1

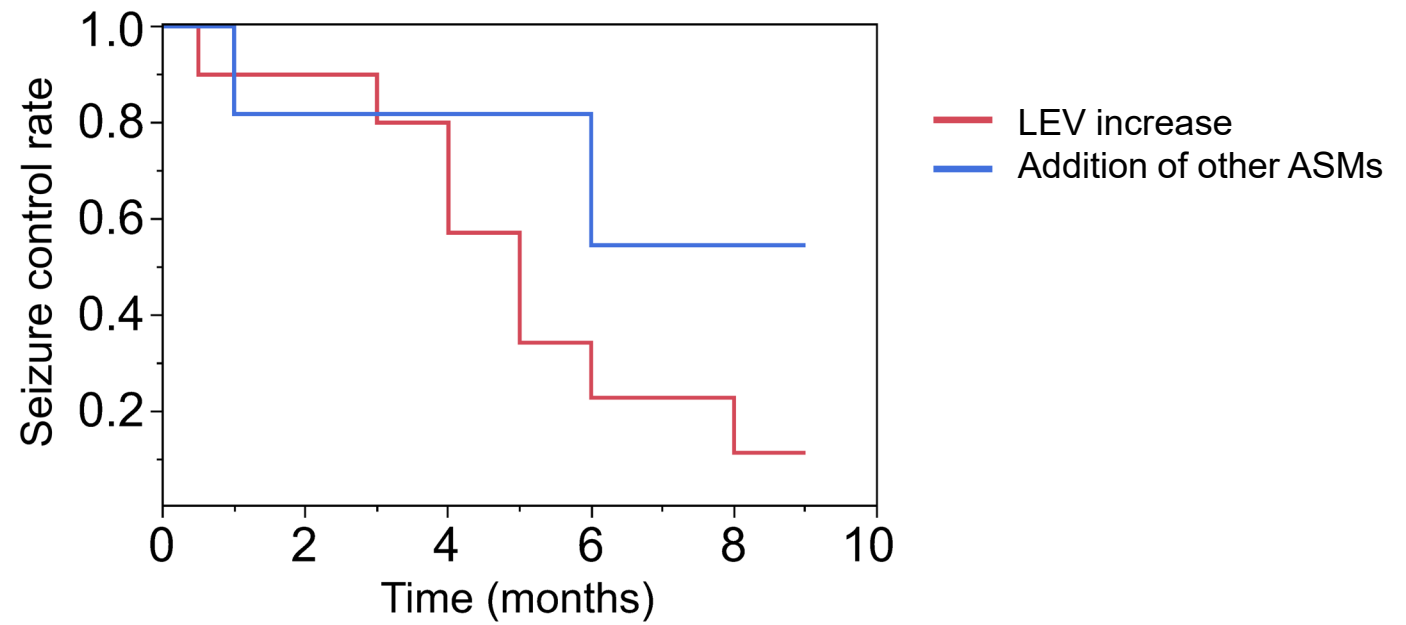

Supplementary Figure 2

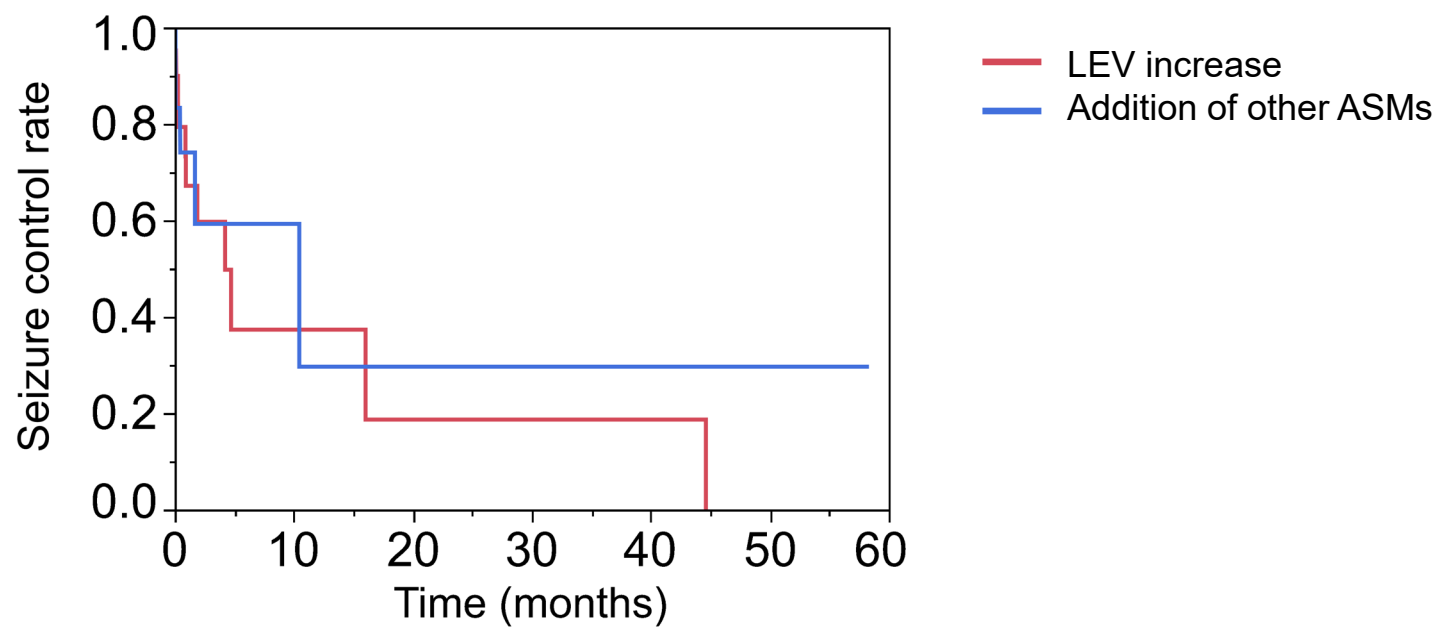

Supplement: Supplementary file 1 — Supplementary Material 1 [file 12885_2023_11273_MOESM1_ESM.pdf]
