# Supplementary material for: Seizure control by adding on other anti-seizure medication on seizure during levetiracetam administration in patients with glioma-related epilepsy
Source: BMC Cancer. 2023 Sep 11;23:849. doi: 10.1186/s12885-023-11273-8 (PMC10496310; doi:10.1186/s12885-023-11273-8)
Supplement: Supplementary file 2 — Supplementary Material 2 [file 12885_2023_11273_MOESM2_ESM.docx]

**Supplementary Figure 1. Seizure control rates after first and second seizures in patients receiving LEV as first-line treatment, by difference in treatment**

Patients were combined after first and second seizures. Treatment is indicated by red line for “LEV increase” and blue line for “addition of other ASMs”. "Addition of other ASMs” group tended to have better control rates, but the difference was not significant (*p* = 0.163).

**Supplementary Figure 2. Seizure control rates after first and second seizures in patients receiving LEV as non-first-line treatment, by difference in treatment**

Patients were combined after first and second seizures. Treatment is indicated by red line for “LEV increase” and blue line for “addition of other ASMs”. There was no significant difference in control rates between the two groups (*p* = 0.602).
