# Supplementary material for: Seizure control by adding on other anti-seizure medication on seizure during levetiracetam administration in patients with glioma-related epilepsy
Source: BMC Cancer. 2023 Sep 11;23:849. doi: 10.1186/s12885-023-11273-8 (PMC10496310; doi:10.1186/s12885-023-11273-8)
Supplement: Supplementary file 3 — Supplementary Material 3 [file 12885_2023_11273_MOESM3_ESM.docx]

**Supplementary table 1. ASMs added for seizure occurrence**

| ASMs | First-line LEV | Non-first-line LEV |
| --- | --- | --- |
| Number of patients | 12 | 12 |
|  |  |  |
| PER | 6 | 2 |
| CLB | 2 | 4 |
| VPA | 2 | 1 |
| CBZ | 1 | 1 |
| PHT | 1 | 0 |
| ZNS | 0 | 2 |
| LTG | 0 | 1 |
| PB | 0 | 1 |

ASM, antiseizure medication; LEV, levetiracetam; PER, perampanel; CLB, clobazam; VPA, valproic acid; CBZ, carbamazepine; PHT, phenytoin; ZNS, zonisamide; LTG, lamotrigine; PB, phenobarbital.
