# Supplementary material for: Seizure control by adding on other anti-seizure medication on seizure during levetiracetam administration in patients with glioma-related epilepsy
Source: BMC Cancer. 2023 Sep 11;23:849. doi: 10.1186/s12885-023-11273-8 (PMC10496310; doi:10.1186/s12885-023-11273-8)
Supplement: Supplementary file 4 — Supplementary Material 4 [file 12885_2023_11273_MOESM4_ESM.docx]

**Supplementary table 2. Adverse events for each ASM**

| Adverse events | LEV | ZNS | VPA | | CBZ | | PHT | | CLB | | PER | | LTG | | GBP | |
| --- | --- | --- | --- | --- | --- | --- | --- | --- | --- | --- | --- | --- | --- | --- | --- | --- |
| Number of patients | 34/160  (21.3%) | 30/93  (32.3%) | 14/56  (25.0%) | 8/37  (21.6%) | | 3/31  (9.7%) | | 10/60  (16.7%) | | 4/19  (21.1%) | | 4/17  (23.5%) | | 2/12  (16.7%) | |  |
| Number of symptoms | 37 | 32 | 15 | 9 | | 3 | | 10 | | 4 | | 5 | | 2 | |  |
|  |  |  |  |  | |  | |  | |  | |  | |  | |  |
| Drowsiness / wobbly | 14 (8.8) | 3 (3.2) | 2 (3.6) | 2 (5.4) | | 0 | | 6 (10.0) | | 4 (21.1) | | 0 | | 0 | |  |
| Cytopenia | 7 (4.4) | 6 (6.5) | 6 (10.7) | 1 (2.7) | | 0 | | 2 (3.3) | | 0 | | 1 (5.9) | | 0 | |  |
| Drug rash | 4 (5.6) | 17 (18.3) | 2 (3.6) | 5 (13.5) | | 1 (3.2) | | 1 (3.3) | | 0 | | 3 (17.6) | | 1 (8.3) | |  |
| Fatigue / feeling bad | 4 (2.5) | 2 (2.2) | 0 | 0 | | 0 | | 1 (1.7) | | 0 | | 0 | | 1 (8.3) | |  |
| Psychological symptoms (irritation / delirium) | 2 (1.3) | 0 | 0 | 0 | | 0 | | 0 | | 0 | | 0 | | 0 | |  |
| Joint pain | 2 (1.3) | 0 | 0 | 0 | | 0 | | 0 | | 0 | | 0 | | 0 | |  |
| Anorexia / digestive symptoms | 3 (1.9) | 2 (2.2) | 1 (1.8) | 0 | | 1 (3.2) | | 0 | | 0 | | 0 | | 0 | |  |
| Hepatic dysfunction | 1 (0.6) | 0 | 2 (3.6) | 1 (2.7) | | 1 (3.2) | | 0 | | 0 | | 0 | | 0 | |  |
| Extrapyramidal symptoms / neurological symptoms | 0 | 2 (2.2) | 2 (3.6) | 0 | | 0 | | 0 | | 0 | | 0 | | 0 | |  |
| Fever | 0 | 0 | 0 | 0 | | 0 | | 0 | | 0 | | 1 (5.9) | | 0 | |  |

ASM, antiseizure medication; LEV, levetiracetam; ZNS, zonisamide; VPA, valproic acid; CBZ, carbamazepine; PHT, phenytoin; CLB, clobazam; PER, perampanel; LTG, lamotrigine; GBP, gabapentin.

*Numbers in parentheses represent the percentage of cases with each adverse event among all patients using each ASM.
